# Obstructive sleep apnea syndrome and risk of renal impairment: a systematic review and meta-analysis with trial sequential analysis

**DOI:** 10.1007/s11325-020-02090-5

**Published:** 2020-05-21

**Authors:** Tongtong Liu, Yongli Zhan, Yuyang Wang, Qian Li, Huimin Mao

**Affiliations:** Guang’anmen Hospital, China Academy of Chinese Medical Sciences, No.5, Bei Xian Ge Street, Xicheng District, Beijing, 100053 China

**Keywords:** Obstructive sleep apnea syndrome, Renal damage, Cystatin C, Meta-analysis, Trial sequential analysis

## Abstract

**Background:**

Obstructive sleep apnea syndrome (OSAS) is associated with a variety of systemic diseases. Among patients with chronic kidney diseases (CKD), the prevalence of OSAS is high. OSAS can induce progression of CKD. However, whether or not OSAS can cause renal damage in healthy people is not clear. Thus, the purpose of this meta-analysis was to elucidate whether or not there was an association between OSAS and early renal damage.

**Methods:**

PubMed, Embase Database, Cochrane Library, Web of Science, China National Knowledge Infrastructure, China Biology Medicine Database, Chinese Scientific Journals Database, and Wanfang Database were searched systematically. The relative risk (RR), weighted mean difference (WMD), and 95% confidence intervals (CI) were used to evaluate the relationship between OSAS and early renal damage. Funnel plot and Egger’s test were used to evaluate publication bias, and trial sequential analysis (TSA) was employed to verify the sufficiency of the research conclusions.

**Results:**

A total of 18 studies were analyzed comprising 4,567 participants. Compared with the healthy control group, levels of cystatin C (MD = 0.530, 95% CI 0.423, 0.637, *P* < 0.01) and proteinuria in patients with OSAS were significantly increased, while the levels of estimated glomerular filtration rate (eGFR) (MD = − 0.194, 95% CI − 0.268, − 0.121, *P* < 0.01) were significantly decreased. Furthermore, patients with OSAS also had an increased risk of CKD. Subgroup analysis showed that compared with patients without OSAS, the level of serum cystatin C in patients with OSAS was significantly increased independent of hypertension and diabetes, and the eGFR was significantly decreased in patients with moderate to severe OSAS and comorbid hypertension and/or diabetes.

**Conclusion:**

In this meta-analysis, OSAS was associated with a higher risk of early renal damage. Patients with OSAS and comorbid hypertension and/or diabetes appear to suffer from severe renal damage.

## Introduction

Obstructive sleep apnea syndrome (OSAS) is one of the common forms of sleep disorders, characterized by recurrent episodes of apnea and hypopnea due to obstruction of the upper airway during sleep [1]. Evidence obtained from clinical study supports that the risk of several comorbidities, including cardiovascular events [2, 3], stroke [4], diabetes [5], and chronic kidney disease [6], among patients with OSAS is high. It is reported that more than 57% patients with CKD are suffering from OSAS [7], accompanied with unfavorable prognosis and an increase in mortality [8, 9].

Serum cystatin C and urinary microalbuminuria are much earlier and sensitive biomarkers for renal impairment [10]. Emerging studies have found that OSAS was significantly associated with increased cystatin C and microalbuminuria excretion in patients without CKD [11, 12]. Studies related to the relationship between OSAS and potential renal impairment suggest opposite results [13]. Therefore, a meta-analysis is needed for further assessment. The purpose of the current study was to evaluate the possible risk of renal impairment in OSAS patients, which might help to offer novel strategies for treatment and risk assessment.

## Materials and methods

This meta-analysis was conducted based on Preferred Reporting Items for Systematic Review and Meta-Analyses (PRISMA) checklist [14].

### Literature retrieval strategy

**Fig. 1:**
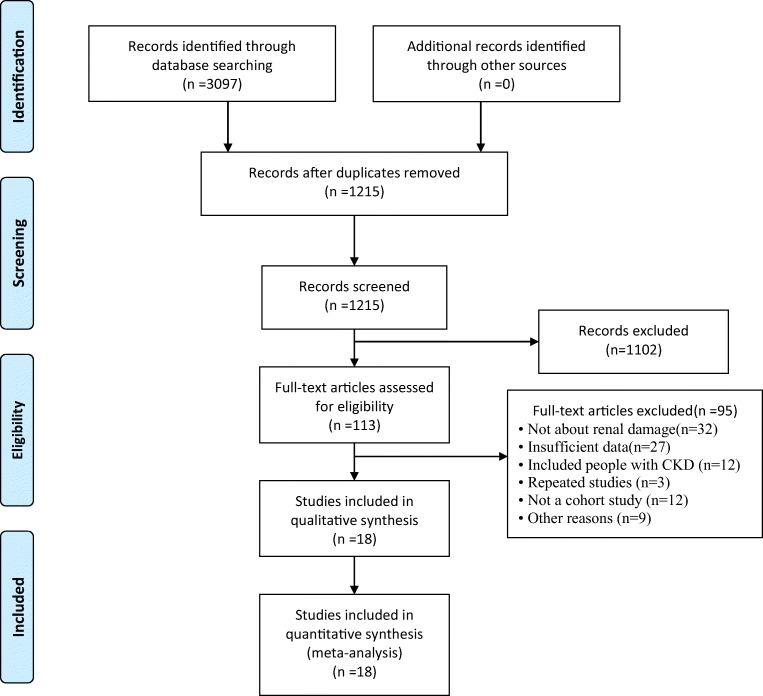
Flow chart for the process of literature selection

PubMed, EMBASE Database, Cochrane Library, Web of Science, China National Knowledge Infrastructure (CNKI), China Biology Medicine Database (CBM), Chinese Scientific Journals Database (VIP), and Wanfang Database were independently searched by two researchers (from database inception to December 2019). The MeSH and keywords adjusted according to the characteristics of different databases were as follows: obstructive sleep apnea syndrome, sleep apnea, apnea-hypopnea index (AHI), sleep-associated breathing disorder, OSAS, OSA, SA, renal damage, renal dysfunction, serum cystain C, and microalbuminuria. This study includes English and Chinese. The flowchart for the process of literature selection is shown in Fig. 1.

### Inclusion and exclusion criteria

Clinical studies eligible for this meta-analysis included cohort studies and case-control studies. Studies were considered eligible if they (1) enrolled participants with OSAS, (2) enrolled participants without CKD, (3) compared the risk of renal damage with healthy control group, and (4) provided information on any of the primary or secondary outcomes.

### Data extraction and quality assessment

Two researchers (HM MAO and Q LI) screened the literature independently according to the inclusion and exclusion criteria, and rescreened and extracted data for the remaining retrieval results. The literature in dispute or unable to extract data needs to be discussed with the third researcher (YL ZHAN). The content of the extracted data includes the following: the basic information, the sample size of participants, the assessment tools of OSAS, and the outcome index. The Newcastle–Ottawa Scale (NOS) provided by Cochrance was used to evaluate the quality of the included studies.

### Outcomes

The primary outcome of this meta-analysis was serum cystatin C. The secondary outcomes were (1) eGFR (as assessment by study authors), (2) incidence of CKD (defined as eGFR < 60 ml/min/1.73 m^2^), and (3) progression of albuminuria-related indexes (including albumin/creatinine ratio, incidence of microalbuminuria, and the levels of microalbuminuria).

### Statistical analyses

The meta-analysis was carried out with STATA 14.0, and the trial sequential analysis (TSA) was carried out with TSA0.9. If the study data was enumeration data, the relative risk (RR) and 95% confidence intervals (CI) were used as the statistical effect quantity; otherwise, the standardized mean difference (SMD) and 95%CI were used as the statistical effect quantity to evaluate the relationship between OSAS and renal damage. Chi-squared test and I^2^ statistic were used to test the heterogeneity of the included studies. When *P* > 0.05 and I^2^ < 50%, fixed effect model was used for meta-analysis; otherwise, random effect model was used. At the same time, funnel chart and Egger’s test were used to evaluate the potential publication bias. Finally, TSA of the main results were analyzed in order to evaluate the adjusted statistical significance threshold and the required information size (RIS), so as to correct and reduce the random error.

## Results

A total of 3097 related literatures were retrieved. After removing the repetition, reading title and abstract, and further reading the full text, 18 cross-sectional studies [11, 12, 15–30] were included and the NOS score of each study were all above 5. A total of 4567 participants were included: 3472 with OSAS and 1095 in healthy control group. Among the included OSAS patients, 574 were with mild OSAS, 618 with moderate OSAS, 698 with severe OSAS, and other 1582 with unidentified stage of OSAS (the severity of OSAS was assessed by study authors). These studies are conducted in different countries, such as USA [11, 17, 19], China [15, 16, 18, 23, 24, 28, 30], Greece [12, 20], Turkey [21, 22, 26], Germany [25], and Australia [27]. Six studies [12, 16, 18, 20, 21, 28] controlled the confounding factors such as diabetes and high blood pressure. The characteristics of the included studies are shown in Table 1.

**Table 1:** The characteristics of the included studies

Study	Country	No. of participants	No. of comorbidity		No. of subjects (case/control)	Sex male(%)	Age (year)	Assessment of OSAS	Outcome	Quality score
			DM	HTN						
Agrawal2009 [11]	USA	91	31	55	36/55	27.1%	44.9 ± 9.9	PSG, AHI ≥ 5	③④	8
Voulgaris2018 [12]	Greece	96	0	0	32/64	79.17%	50.7 ± 11.98	PSG, AHI ≥ 5	①②	7
Chen2015 [15]	China	457	0	457	106/351	64.11%	20 ~ 78	PSG, AHI ≥ 10	①④	7
Chen2019 [16]	China	90	0	0	17/73	NA	42.50 ± 11.46	PSG, AHI ≥ 10	①②	7
Canales2011 [17]	USA	507	66	359	120/387	100%	76.0 ± 5.3	PSG, RDI ≥ 5	② ③④	8
Zhang2013 [18]	China	98	0	0	23/75	100%	32.5 ± 5.19	PSG, AHI ≥ 5	①②	6
Faulx2007 [19]	USA	496	172	63	233/263	44.4%	44.5 ± 17.3	PSG, AHI ≥ 5	②③	7
Archontogeorgis 2016 [20]	Greece	84	0	0	20/64	80.95%	51.69 ± 12.71	PSG, AHI ≥ 5	①②	7
Ursavas2008 [21]	Turkey	46	0	0	11/35	100%	45.12 ± 10.67	PSG, AHI ≥ 5	③	6
Bulcun2015 [22]	Turkey	124	0	NA	26/98	74.19%	48.38 ± 11.79	PSG, AHI ≥ 5	②③④	6
Song2019 [23]	China	487	49	331	54/433	84.8%	40.08 ± 11.6	PSG, AHI ≥ 5	①②	6
Zeng2017 [24]	China	109	8	21	26/83	100%	45.72	PSG, AHI ≥ 5	②③	6
Yayan2017 [25]	Germany	382	NA	NA	19/363	69.63%	63.75 ± 13.85	PSG, AHI ≥ 5	④	5
Uyar2015 [26]	Turkey	696	0	275	62/634	68.1%	51.09 ± 11.82	PSG, AHI ≥ 5	③	6
Robert2017 [27]	Australia	489	NA	NA	237/252	NA	NA	PSG, AHI ≥ 10	④	8
Chou2011 [28]	Taiwan, China	40	0	0	3/37	83%	44.8 ± 8.6	PSG, AHI ≥ 5	⑤	7
Kanbay2012 [29]	Turkey	175	30	34	25/150	66.86%	53.94 ± 12.16	PSG, AHI ≥ 5	③	7
Hou2016 [30]	China	100	0	100	45/55	60%	55.04 ± 11.09	PSG, AHI ≥ 5	①	7

*PSG*, polysomnography; *DM*, diabetes mellitus; *HTN*, hypertension; *AHI*, apnea-hypopnea index; *outcome*, ① cystatin C; ② eGFR; ③ ACR; ④ albuminuria; ⑤ new-onset CKD

### Serum cystatin C

**Fig. 2:**
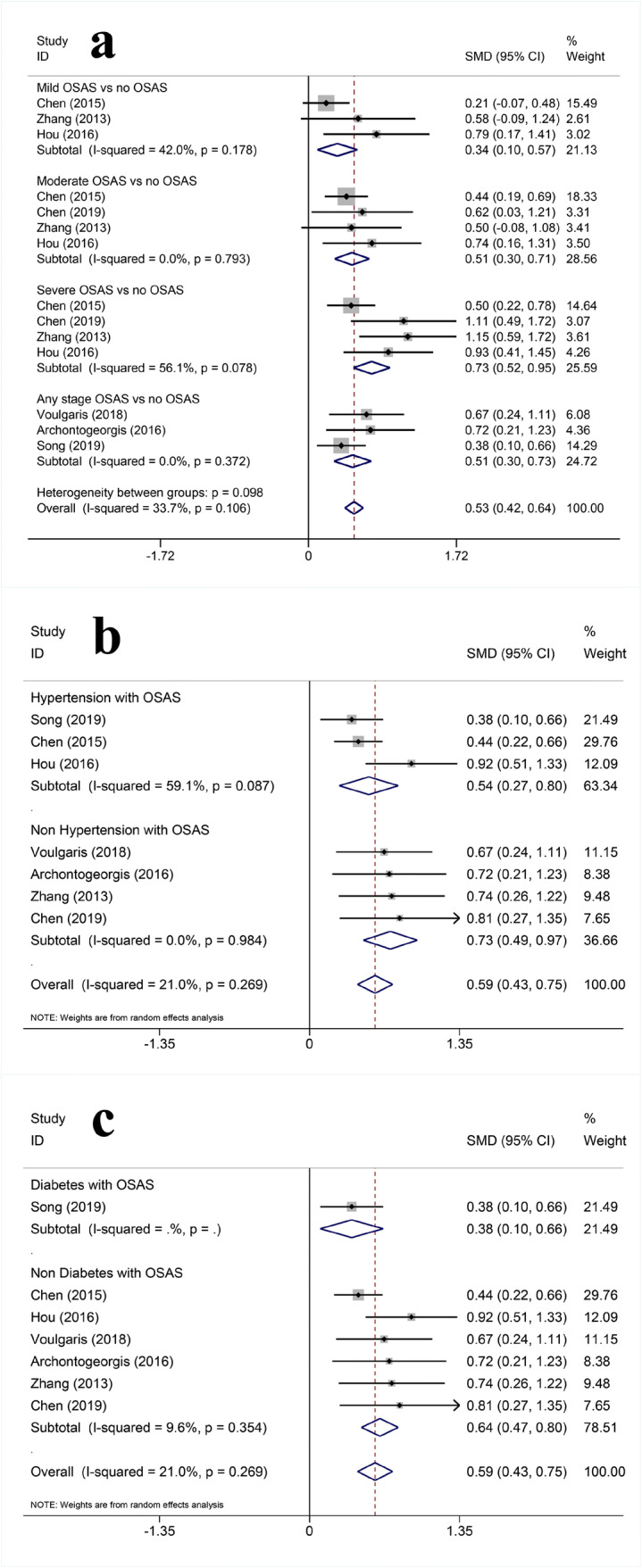
Forest plots of the association between serum OSAS and cystatin C. **a** Relationship between severity of OSAS and cystatin C. **b** Relationship between hypertension and cystatin C in OSAS patients. **c** Relationship between diabetes and cystatin C in OSAS patients

Evidence from 7 studies [12, 15, 16, 18, 20, 23, 30] (enrolling 1412 individuals) suggests that serum cystatin C was closely related to OSAS (MD = 0.530, 95%CI (0.423, 0.637), *P* < 0.01). The results of subgroup analysis showed that the serum cystatin C increased significantly in mild, moderate and severe OSAS The level of cystatin C was positively correlated with the severity of OSAS, whether they have hypertension and diabetes or not (Fig. 2 and Table 2).

**Table 2:** Meta-analysis of association between OSAS and renal damage

Outcome	Subgroup	No. of studies	Heterogeneity		Effect size		
			*P*	I^2^	Pooled SMD	95%CI	*P*
Serum cystatin C	Mild OSAS vs. no OSAS	3	0.178	42.0%	0.335	0.102, 0.569	< 0.01
	Moderate OSAS vs. no OSAS	4	0.793	0.0%	0.506	0.305, 0.706	< 0.01
	Severe OSAS vs. no OSAS	4	0.078	56.1%	0.734	0.522, 0.946	< 0.01
	Any stage OSAS vs. no OSAS	3	0.372	0.0%	0.513	0.297, 0.728	< 0.01
	Overall	7	0.106	33.7%	0.530	0.423, 0.637	< 0.01
	Hypertension with OSAS vs. hypertension with no OSAS	3	0.087	59.1%	0.535	0.268, 0.802	< 0.01
	Non-hypertension with OSAS vs. non-hypertension with no OSAS	4	0.984	0.0%	0.730	0.487, 0.974	< 0.01
	Overall	7	0.269	21.0%	0.592	0.433, 0.751	< 0.01
	Diabetes with OSAS vs. diabetes with no OSAS	1	/	/	0.381	0.097, 0.665	< 0.01
	Non-diabetes with OSAS vs. non-diabetes with no OSAS	6	0.354	9.6%	0.636	0.470, 0.802	< 0.01
	Overall	7	0.269	21.0%	0.592	0.433, 0.751	< 0.01
eGFR	Mild OSAS vs. no OSAS	6	0.445	0.0%	− 0.122	− 0.262, 0.018	0.087
	Moderate OSAS vs. no OSAS	7	0.121	40.6%	− 0.218	− 0.362, − 0.073	< 0.01
	severe OSAS vs. no OSAS	7	0.411	1.8%	− 0.341	− 0.488, − 0.193	< 0.01
	Any stage OSAS vs. no OSAS	5	0.077	52.6%	− 0.096	− 0.250, 0.059	0.226
	Overall	13	0.057	33.1%	− 0.194	− 0.268, − 0.121	< 0.01
	Hypertension with OSAS vs. hypertension with no OSAS	6	0.057	53.3%	− 0.224	− 0.387, − 0.061	0.007
	Non-hypertension with OSAS vs. non-hypertension with no OSAS	4	0.097	52.5%	0.006	− 0.343, 0.354	0.975
	Overall	10	0.016	55.8%	− 0.159	− 0.317, − 0.002	0.047
	Diabetes with OSAS vs. diabetes with no OSAS	5	0.032	62.2%	− 0.237	− 0.439, − 0.035	0.021
	Non-diabetes with OSAS vs. non-diabetes with no OSAS	6	0.180	34.2%	− 0.042	− 0.253, 0.169	0.699
	Overall	11	0.021	52.3%	− 0.149	− 0.296, − 0.001	0.049

### eGFR

**Fig. 3:**
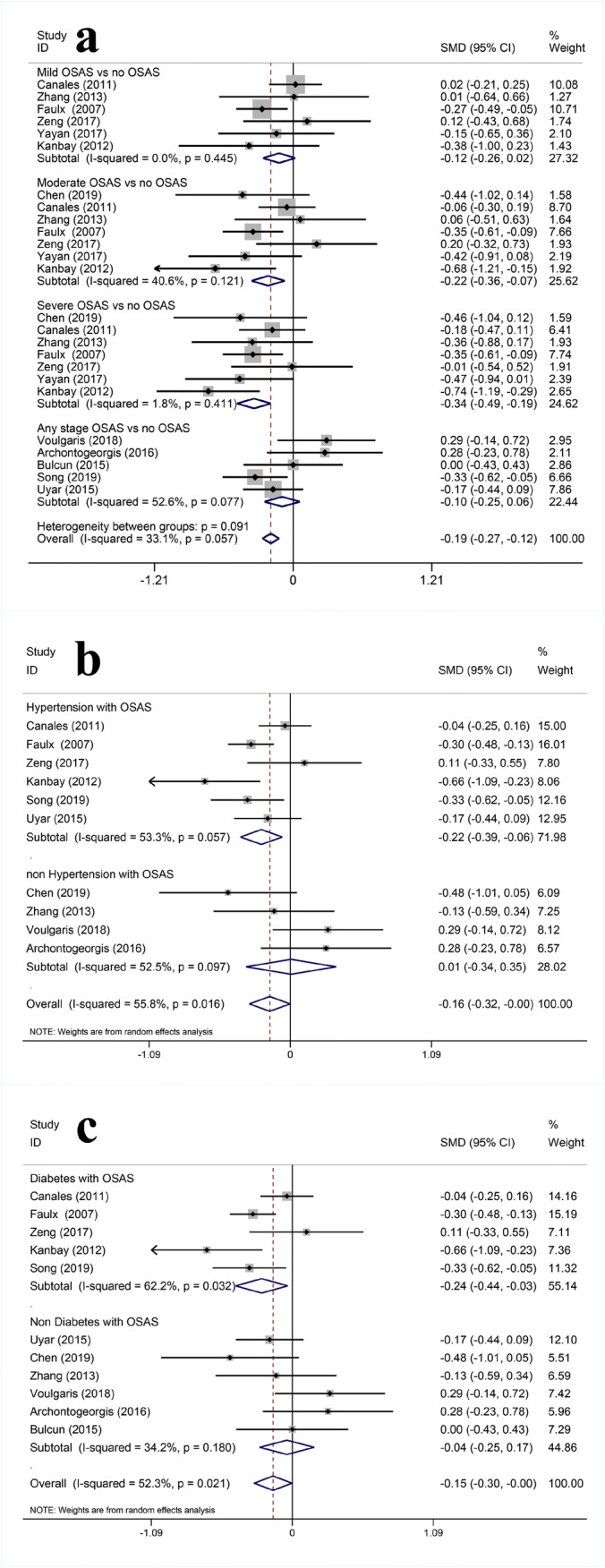
Forest plots of the association between serum OSAS and eGFR. **a** Relationship between severity of OSAS and eGFR. **b** Relationship between hypertension and eGFR in OSAS patients. **c** Relationship between diabetes and eGFR in OSAS patients

We included 12 studies [12, 16–20, 22–26, 29] to explore the relationship between eGFR and OSAS, including 3344 individuals. Three studies [24–26] used the Chronic Kidney Disease Epidemiology Collaboration (CKD-EPI) formula to estimate eGFR; 6 studies [12, 16–18, 20, 22] used the Modification of Diet in Renal Disease equation (MDRD); 1 study [29] used the Cockroft-Gault equation; 1 study [19] calculated eGFR from serum cystatin C levels and sex-corrected; 1 study [23] used the combined creatinine-cystatin C equation. The results show that OSAS was significantly correlated with decreased eGFR (MD = − 0.194, 95%CI (− 0.268, − 0.121), *P* < 0.01). The results of subgroup analysis showed that the decrease of eGFR is related to moderate and severe OSAS. The levels of eGFR decreased significantly in OSAS patients with hypertension and diabetes, but this phenomenon was not found in those who were not diagnosed with hypertension or diabetes. (Fig. 3 and Table 2).

### New-onset CKD

**Fig. 4:**
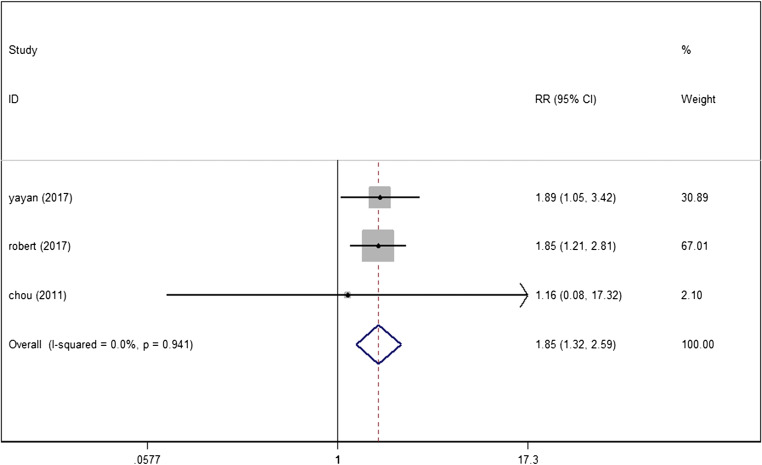
Forest plot of the association between serum OSAS and new-onset CKD

We found that other 3 studies [25, 27, 28] including 911 persons reported a significantly increased risk of CKD in OSAS (RR = 1.847, 95%CI (1.315, 2.593), *P* < 0.01) (Fig. 4).

### Albuminuria

Additionally, 7 studies [11, 15, 17, 19, 21, 22, 24] found that there was a certain relationship between OSAS and albuminuria, 3 [17, 22, 24] of which found that patients with OSAS had higher ACR, 4 [11, 17, 19, 22] of which found that patients with OSAS were more likely to have microalbuminuria (MAU), and 2 [15, 21] of which found that patients with OSAS had higher levels of microalbuminuria (Table 3).

**Table 3:** Meta-analysis of association between OSAS and albuminuria

Outcome	No. of studies	No. of participants	Heterogeneity		Effect model	Effect size		
			*P*	I^2^		WMD/RR	95%CI	*P*
ACR								
Mild OSAS vs. no OSAS	2	352	0.594	0%	Random	0.422	0.207, 0.637	< 0.01
Moderate OSAS vs. no OSAS	2	306	0.688	0%	Random	0.886	0.651, 1.121	< 0.01
Severe OSAS vs. no OSAS	2	250	0.355	0%	Random	1.072	0.801, 1.342	< 0.01
Any stage OSAS vs. no OSAS	1	124	/	/	Random	0.375	− 0.060, 0.809	0.091
Overall	3	740	0.003	69.2%	Random	0.712	0.582, 0.843	< 0.01
No. of MAU	4	667	0.578	0%	Fixed	2.316	1.480, 3.623	< 0.01
Levels of MAU	2	502	0.00	88.5%	Random	0.432	0.276, 0.676	< 0.01

### Publication bias

**Fig. 5:**
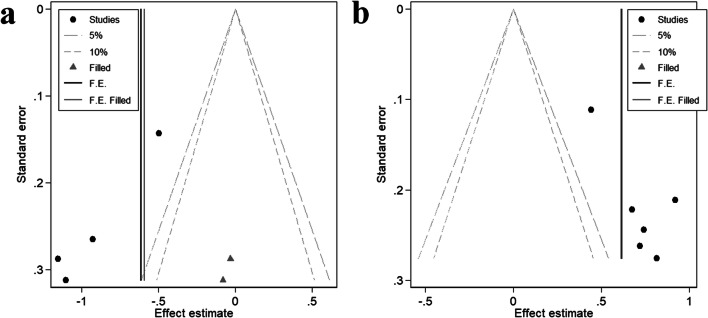
Additional contour funnel plot. **a** Cystatin C of severe OSAS vs no OSAS. **b** Cystatin C of non-diabetes patients with OSAS vs no OSAS

Funnel plot and Egger’s test were used to analyze the publication bias of the main indexes. Except for the subgroup of cystatin C in severe OSAS and cystatin C in OSAS without diabetes, there was no significant publication bias in other subgroup (Table 4). In order to eliminate the influence of publication bias on meta-analysis, the trim and fill analysis were carried out in subgroup of cystatin C in severe OSAS and cystatin C in OSAS without diabetes. The pooled SMD (95%CI) after trim and fill analysis were 0.593 (0.782, − 0.405) and 0.617 (0.465, 0.768) (Fig. 5).

**Table 4:** Egger’s test of outcome

Outcome	*t*	*P*	95%CI
Serum cystatin C			
Mild OSAS vs. no OSAS	− 2.97	0.207	− 13.346, 8.284
Moderate OSAS vs. no OSAS	− 2.04	0.179	− 3.294, 1.178
Severe OSAS vs. no OSAS	− 9.19	0.012	− 5.717, − 2.070
Any stage OSAS vs. no OSAS	− 5.65	0.111	− 10.281, 3.951
HTN with OSAS vs. HTN with no OSAS	− 1.49	0.376	− 41.470, 32.754
Non-HTN with OSAS vs. non-HTN with no OSAS	− 2.54	0.126	− 5.693, 1.465
Diabetes with OSAS vs. diabetes with no OSAS	/	/	/
Non-diabetes with OSAS vs. non-diabetes with no OSAS	− 3.50	0.025	− 4.321, − 0.497
eGFR			
Mild OSAS vs. no OSAS	− 0.63	0.566	− 22.362, 14.139
Moderate OSAS vs. no OSAS	− 0.33	0.756	− 11.788, 9.120
Severe OSAS vs. no OSAS	0.78	0.473	− 5.835, 10.886
Any stage OSAS vs. no OSAS	− 0.73	0.516	− 240.845, 150.567
HTN with OSAS vs. HTN with no OSAS	1.08	0.343	− 22.584, 51.134
Non-HTN with OSAS vs. non-HTN with no OSAS	− 0.54	0.641	− 10.255, 7.956
Diabetes with OSAS vs. diabetes with no OSAS	0.63	0.573	− 33.744, 50.425
Non-diabetes with OSAS vs. non-diabetes with no OSAS	− 0.78	0.480	− 195.660, 109.927
Incident CKD	1.97	0.299	− 1.986, 2.713
ACR	0.59	0.574	− 2.913, 4.900
No. of MAU	− 0.71	0.550	− 6.489, 4.646

*HTN*, hypertension

### Trial sequential analysis

**Fig. 6:**
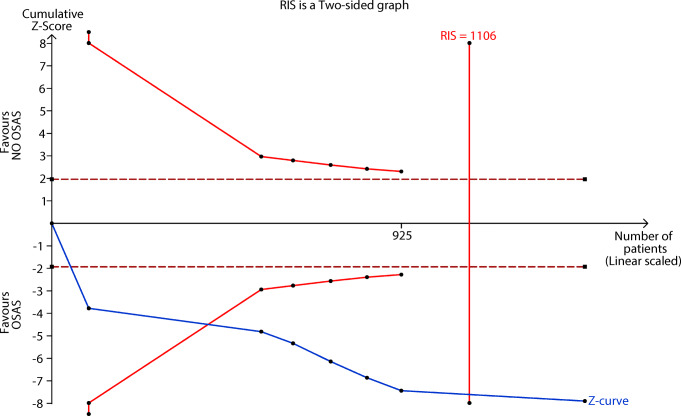
TSA of the relationship between cystatin C and OSAS

We conducted a TSA of seven studies on the relationship between cystatin C and OSAS We set type I error as 5%, and the power as 80%. Based on this analysis, the accumulated Z-curve crossed the traditional and TSA thresholds and reaches the RIS (1106) (Fig. 6), which indicates that the result of meta-analysis is more robust and persuasive.

## Discussion

CKD, a public health problem, is affecting about 8%  -16% people around the world and brings heavy economic burden [31]. Diabetes, hypertension, and obesity are traditional risk factors that affect the occurrence and progress of CKD. In recent years, emerging risk factors are being recognized including OSAS [32]. Studies have found that an increasing number of patients with OSAS may suffer from mild CKD [33, 34]. Sklar [35] reported the association between severe OSA and proteinuria. Then, through the treatment of OSAS, proteinuria was relieved. Coincidentally, Daskalopoulou [36] found that compared with healthy people, the albuminuria excretion of OSAS patients increased during sleep. A survey in Japan found that the incidence of CKD in OSAS was much higher than that in people without OSAS (30.5% vs. 9.1%) ^[38]^. Chou [28] found a significant correlation between the severity of OSAS and renal impairment. Similarly, a large cohort study of US veterans by Molnar [37] found that patients diagnosed with OSAS had a faster decline in renal function.

OSAS may result in the occurrence and development of CKD by aggravating traditional risk factors such as hypertension, diabetes, and obesity [38]. On the other hand, hypoxia of renal tissue is considered as an important mediator for the occurrence and progression of CKD [39]. OSAS results in activated hypoxia-inducible factors (HIFs) which causes cascade reactions, such as inflammatory response and endothelial damage [40], ultimately leading to renal damage and dysfunction. Furthermore, hypoxia can induce renal damage by activating sympathetic nervous system or renin-angiotensin-aldosterone system (RAAS), and eventually progresses into CKD [41, 42]. Renal damage caused by OSAS is often manifested as nocturia, microalbuminuria, and renal dysfunction. Consequently, many studies have found that continuous positive airway pressure (CPAP) therapy can effectively alleviate the progress of CKD [43], reverse the decline of eGFR [44], and reduce the urinary albumin excretion (UAE) [45].

Findings from this meta-analysis suggest that the levels of serum cystatin C and proteinuria in patients with OSAS were significantly higher, and the levels of eGFR were obviously lower than those in healthy people without OSAS. Similarly, compared with mild to moderate OSAS patients, the levels of serum cystatin C, proteinuria, and eGFR in patients with severe OSAS showed the same changes as the above results. Furthermore, our meta-analysis also found an increased risk of CKD in OSAS patients.

Hypertension and diabetes are common risk factors in OSAS and CKD. In order to reduce the potential impact of hypertension and diabetes on this study, we conducted a subgroup analysis of serum cystatin C and eGFR based on whether the study included patients with hypertension and diabetes. The results showed that with or without hypertension and diabetes, the levels of cystatin C increased in OSAS patients, and there was no significant difference between the two groups. Conversely, for eGFR, OSAS with hypertension and diabetes decreased significantly.

Serum cystatin C and urinary microalbumin are considered to be more sensitive markers in the diagnosis of early renal damage. Viazzi [46] reported that 50% of patients with OSAS may progress to end stage renal disease (ESRD) when they have abnormal urinary microproteins and renal function. Therefore, this meta-analysis shows that OSAS can induce potential kidney damage.

There are some limitations in this study. First, the amount of literature included in this study is small, which may affect the accuracy of our analysis results. Second, some studies have found that obesity and male are more likely to be suffering from OSAS [47], while BMI and gender will affect renal function at the same time. Unfortunately, we cannot exclude the impact of BMI and gender on this study, which may affect the validity of the main results. In addition, the renal damage caused by OSAS is long lasting. However, the studies included in our meta-analysis were cross-sectional, and more cohort studies are needed to confirm our conclusions.

## Conclusion

This meta-analysis found that OSAS was associated with a higher risk of early renal damage, especially in the patients with hypertension and/or diabetes. The result of TSA verifies the credibility and stability of the conclusions. Patients with OSAS should evaluate renal function regularly aiming to decrease the risk of developing into CKD. Notably, this meta-analysis requires more prospective cohort studies to reduce the impact of confounding factors, so as to clarify whether OSAS is an independent risk factor for renal damage.
